# Ultrasound-Assisted and Ultrasound-Guided Thoracentesis: An Educational Review

**DOI:** 10.3390/diagnostics14111124

**Published:** 2024-05-29

**Authors:** Andrea Boccatonda, Chiara Baldini, Davide Rampoldi, Giacomo Romani, Antonio Corvino, Giulio Cocco, Damiano D’Ardes, Orlando Catalano, Luigi Vetrugno, Cosima Schiavone, Fabio Piscaglia, Carla Serra

**Affiliations:** 1 Interventional, Diagnostic and Therapeutic Ultrasound Unit, Department of Medical and Surgical Sciences, Istituto di Ricovero e Cura a Carattere Scientifico (IRCCS), Azienda Ospedaliero-Universitaria Sant’Orsola Malpighi Hospital, 40138 Bologna, Italy; carla.serra@aosp.bo.it; 2 Department of Medical and Surgical Sciences (DIMEC), University of Bologna, 40126 Bologna, Italy; fabio.piscaglia@unibo.it; 3 Internal Medicine, IRCCS Azienda Ospedaliero-Universitaria Policlinico di Sant’Orsola, 40138 Bologna, Italy; chiara.baldini@studio.unibo.it (C.B.); davide.rampoldi@studio.unibo.it (D.R.); giacomo.romani@studio.unibo.it (G.R.); 4 Movement Sciences and Wellbeing Department, University of Naples Parthenope, 80133 Napoli, Italy; antonio.corvino@uniparthenope.it; 5 Unit of Ultrasound in Internal Medicine, Department of Medicine and Science of Aging, Gabriele d’Annunzio University of Chieti and Pescara, 66100 Chieti, Italy; cocco.giulio@gmail.com (G.C.); cosima.schiavone@gmail.com (C.S.); 6 Institute of “Clinica Medica”, Department of Medicine and Aging Science, Gabriele d’Annunzio University of Chieti and Pescara, 66100 Chieti, Italy; damiano.dardes@unich.it; 7 Radiology Unit, Istituto Diagnostico Varelli, 80126 Naples, Italy; orlando.catalano@istitutovarelli.it; 8 Department of Medical, Oral and Biotechnological Sciences, University of Chieti-Pescara, 66100 Chieti, Italy; luigi.vetrugno@unich.it; 9 Division of Internal Medicine, Hepatobiliary and Immunoallergic Diseases, IRCCS Azienda Ospedaliero-Universitaria di Bologna, 40138 Bologna, Italy

**Keywords:** lung, pleural effusion, thoracentesis, procedure, invasive

## Abstract

Thoracentesis is one of the most important invasive procedures in the clinical setting. Particularly, thoracentesis can be relevant in the evaluation of a new diagnosed pleural effusion, thus allowing for the collection of pleural fluid so that laboratory tests essential to establish a diagnosis can be performed. Furthermore, thoracentesis is a maneuver that can have therapeutic and palliative purposes. Historically, the procedure was performed based on a physical examination. In recent years, the role of ultrasound has been established as a valuable tool for assistance and guidance in the thoracentesis procedure. The use of ultrasound increases success rates and significantly reduces complications. The aim of this educational review is to provide a detailed and sequential examination of the procedure, focusing on the two main modalities, the ultrasound-assisted and ultrasound-guided form.

## 1. Introduction

Thoracentesis is an invasive procedure that is performed to remove fluid through a drainage system temporarily inserted into the pleural cavity [1,2]. The lungs are covered by visceral and parietal pleura, and the space between those two areas is called the pleural space. This space usually contains just a thin layer of fluid, but some conditions such as pneumonia, cancer, congestive heart failure, and many others could cause the developing of excess of fluid [3]. In the presence of pleural effusion, thoracentesis can be performed for diagnostic or therapeutic purposes or both [3]. Thoracentesis can be performed for diagnostic purposes, thus collecting liquid to evaluate the colour or corpuscolarity and to perform chemical-physical investigations, cell counting, cytology, and microbiological examination [1,2,4]. Specifically, thoracentesis may be performed whenever there is a pleural effusion suspected to be of neoplastic nature, to be sampled for cytological examination [1,5]. Moreover, in case of suspected empyema, a thoracentesis may be performed to analyse the fluid to detect the type of germ causing the infection [1,6,7]. On the other hand, the procedure can be performed for therapeutic purposes and to reduce symptoms such as dyspnea and chest pain [1,8,9].

This work aims to summarize the main evidence on the use and methods of performing thoracentesis with ultrasound support. This educational review aims to provide a detailed and sequential examination of the procedure, focusing on the two main modalities, the ultrasound-assisted and ultrasound-guided form. The images we provide are detailed both in terms of the pathological image and the representation of the practical act of carrying out the procedure in the various steps.

A literature search in the MEDLINE (PubMed), Embase, Cochrane Library and Database for Systematic Reviews (CDSR), Google Scholar, and National Institute for Health and Clinical Excellence (NICE) databases that included papers up until March 2024 was performed. Specifically, the search was performed using free text and MeSH terms: thoracentesis, ultrasound, pleural effusion, lung, and invasive procedure.

## 2. Thoracentesis Overview

Originally, thoracentesis was performed without the use of ultrasound guidance, with a procedure mostly known as a “blind procedure” [2,10]. The insertion point of the needle was established based on the objective signs of classical semiotics (the reduction of thoracic expansion, the decrease of tactile vocal fremitus, hypophonesis, and the absence of vesicular murmur) [2,10]. Notably, in the last 20 years, thoracentesis has been performed under ultrasound guidance in most European countries.

The ultrasound guidance significantly increases the likelihood of successful pleural fluid aspiration and reduces the risk of complications (including pneumothorax) [2,11,12].

In a study by Brogi et al., there was a drop in pneumothorax occurrence from 18% in blind thoracentesis to 3% in ultrasound-guided thoracentesis [13]. The diagnostic yield also improves with ultrasound guidance, as evidenced by a decrease from 33% to 0% in “dry taps” [10,13].

Therefore, ultrasound guidance is always recommended for diagnostic aspirations [14]. Therefore, ultrasound allows the method to be performed even in complex settings, e.g., in intensive care or in isolation from an infectious disease [15,16,17].

Thoracentesis with the use of ultrasound can be performed in two ways defined as ultrasound-assisted or ultrasound-guided [14]. In the ultrasound-assisted method, ultrasound is used initially to gather information, but the procedure is performed without real-time ultrasound guidance. On the other hand, the technique is called ultrasound-guided if ultrasound image assistance occurs during the entire procedure itself [12].

## 3. Contraindications to Thoracentesis

Historically, there are three absolute contraindications inducing increased bleeding risk: thrombocytopenia (<50,000/mmc), INR > 1.5, and antiplatelet drugs not discontinued for 5 days [12,18]. The relative contraindications are due to an uncooperative patient, coagulopathy or concurrent anti-coagulation treatment, local infection/cutaneous disease at the proposed puncture site such as cellulitis or herpes zoster, or a partially functioning lung (due to diminished reserve in case of pneumonectomy) [12]. Otherwise, according to the recent BTS guidelines, there are no absolute contraindications to performing thoracentesis, excluding the patient’s lack of consent. The evaluation of coagulation parameters and the correction/suspension of antiplatelet and anticoagulant drugs depends on the setting in which the procedure is performed (emergency vs. routine), thus evaluating the risk/benefit ratio of the procedure [11]. Coagulopathy and the use of anticoagulant drugs can be corrected in a short time with different strategies (specific antagonists or concentrates of prothrombin complexes) and their half-life is generally <24 h or 12 h [12,19].

Direct oral anticoagulant (DOAC) should be stopped 24–48 h before the procedure [20,21]. The guidance is based on the drug’s half-life, the bleeding risk of the procedure, a clinical evaluation of individual risk factors for thrombosis and bleeding, and, in the case of dabigatran, the creatinine clearance (CrCl) [20,21]. DOAC should be resumed one day after a low-risk procedure, and 2–3 days after a high-risk procedure [20,21]. Daily prophylactic heparin should be considered for patients at high risk of venous thrombosis prior to DOAC recommencement [20,21]. Thoracentesis is usually considered a low-risk procedure [21].

In the recent British Thoracic Society (BTS) guideline, no availability of lung ultrasound to identify the procedure site and no safe site for the aspiration of fluid identified on ultrasound have been considered as contraindications to thoracentesis (very small or posterior fluid collections, given the risk to neurovascular bundle lesion) [11].

## 4. Ultrasound-Assisted Procedure

In the assisted thoracentesis, ultrasound is used to obtain information, but the procedure is done without a real-time ultrasound guidance [3]. Since the ultrasound-guided procedure is characterized by several disadvantages, an ultrasound-assisted approach is preferred for most of the pleural procedures [19]. The need of keeping the probe with one hand adds complexity and limits the possibilities of the free hand to touch the rib border and guide the access of the needle [22]. Moreover, the needle is often difficult to identify in real time, as the needle tip and the midpoint appear the same [22]. Paying attention to the needle guide in real time can distract the operator from the execution of the procedure [19].

**Figure 1:**
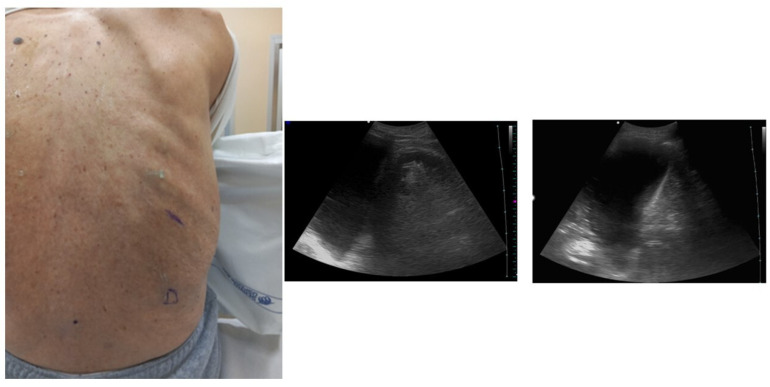
Position of the patient with posterior approach and identification of the landmarks. Ultrasound allows one to identify two landmarks: the apex of the diaphragm at the end of expiration (marked D in the image) and cranially the point (intercostal space) chosen for insertion of the needle. Ultrasound allows one to optimally identify the point where the procedure should be performed. The choice is based on the visualization of the liquid as an anechoic space. The point where the presence of anechoic fluid will be most evident should be the optimal one to perform the procedure. Often at this point the effusion takes on a “V” morphology, delimited on the sides by the diaphragm and the atelectatic lung. The apex of the “V” would represent the specific point where the needle should be inserted and directed.

Firstly, it is relevant to localize the diaphragm [23] (Figure 1). 

Once the kidney is located, the operator moves the probe cranially to pass over the liver or the spleen, and after that the diaphragm can be identified [19]. The apex of the diaphragm at the end of expiration can be marked on the skin to guide the procedure; this point must not be overcome at the caudal level when the thoracentesis is performed to avoid the risk of accidental puncture of the abdominal organs.

**Figure 2:**
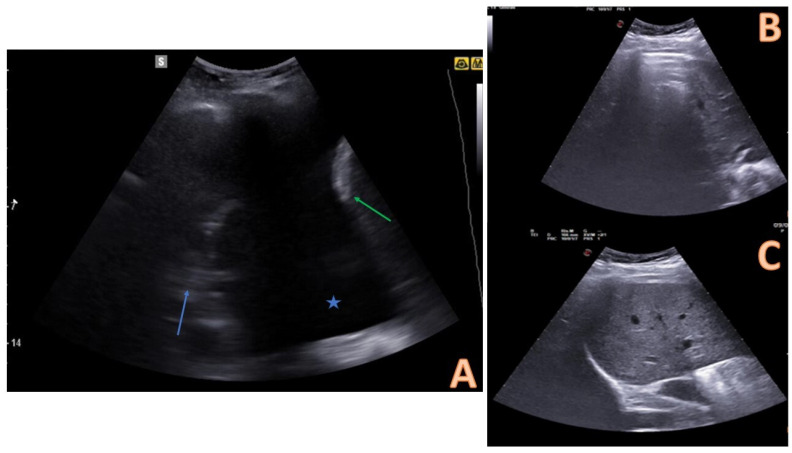
Comparison between pleural effusion (**A**) and healthy lung on ultrasound (**B**,**C**). In (**A**), the pleural effusion is visible below the chest wall and ribs as an anechoic space (blue star). The effusion is delimited inferiorly by a hyperechoic formation that represents the diaphragm (green arrow), and superiorly by an echogenic structure that identifies the atelectatic lung parenchyma (blue arrow). (**B**,**C**) represent the appearance of the healthy lung base on ultrasound: an air interface with A (horizontal) lines is evident. This artefactual image covers the abdominal organ in inspirium (**B**) and uncovers it in expirium (**B**). This sign is called a “curtain sign.” When a pleural effusion is present, the curtain sign cannot be observed.

Subsequently, the pleural effusion should be evaluated by ultrasound qualitatively and quantitatively [23]. On ultrasound, pleural effusion appears like an anechoic area above the diaphragm that can be identified through its anatomic limits (Figure 2) [16]: the diaphragm, the chest wall (at the top of the screen), and the nearby lung. Ultrasound can find a mild amount of fluid <5 mL or 15 mm of size, whereas chest X-ray in a vertical position can detect only a pleural effusion of 150 mL or more [19,24].

According to a study by Lichtenstein, lung ultrasound has a diagnostic accuracy of 93% for the diagnosis of pleural effusion (sensitivity: 92%; specificity: 93%), while the physical examination had a diagnostic accuracy of 61% (sensitivity: 42%; specificity: 90%), and the chest X-ray had a diagnostic accuracy of 47% (sensitivity: 39%; specificity: 85%) [25]. Therefore, lung ultrasound can detect pleural fluid with greater diagnostic accuracy than chest X-ray [19,24].

Notably, in cases of so-called “white hemithorax”, the X-ray cannot carefully distinguish the fluid from the consolidated lung, so it is mandatory to perform an ultrasound before performing an invasive procedure in this type of clinical context, as confirmed by recent BTS guidelines [2]. Moreover, ultrasound is comparable to computed tomography (CT) scanning to quantify and evaluate pleural effusion [23]. 

Indeed, in critical ill patients, lung ultrasound has been shown to display a sensitivity of 94% and a specificity of 97%, compared with CT scans [26].

It is also useful to underline how there may be discrepancies in the quantification of the effusion between ultrasound and CT due to the forced supine position of the patient on CT examination [10,11]. Several studies have tried to validate a predictive formula to estimate the pleural effusion volume, but there is no consensus on an optimum solution [13]. Balik et al. [27] measured the maximal interpleural distance called Sep (between the visceral and parietal pleura) in end-expiration at the lung base and found a good correlation between this measure and the amount of pleural volume. It was suggested that pleural volume could be quantified using the formula: V (ml) = 20 × Sep (mm). However, determining reliable estimation of the effusion volume remains impossible with ultrasound scans because of the complex anatomy of the thorax and the variable size of the thoracic cavity [13]. 

**Figure 3:**
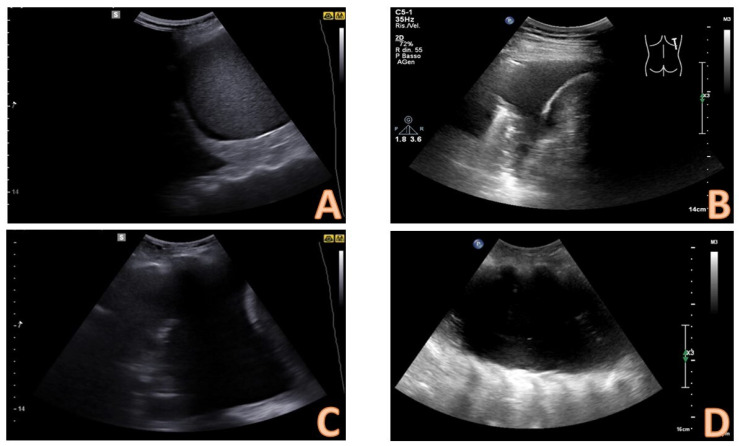
Quantitative evaluation of pleural effusion. This assessment should preferably be performed with the patient seated. It is possible to provide a semi-quantitative estimate of the effusion by counting the number of intercostal spaces occupied by the effusion through longitudinal scans starting from the diaphragm and going up cranially. In image (**A**), there is a minimal effusion, visible only in the costophrenic sinus; in image (**B**), the effusion occupies only an intercostal space and is therefore of a mild degree; in image (**C**). the effusion extends for 2 intercostal spaces and is therefore of moderate degree; in image (**D**), the entire scan is occupied by the effusion with an extension > 3 intercostal spaces and is therefore massive.

Pleural effusions could be evaluated by a semi-quantitative method (Figure 3), thus describing as minimum if the fluid collection is seen only at the costophrenic angle, small if extended only at 1 intercostal space (less than 500 mL), moderate if seen in 2 intercostal spaces (between 500 and 1000 mL), large if greater than 2 intercostal spaces [13]. 

**Figure 4:**
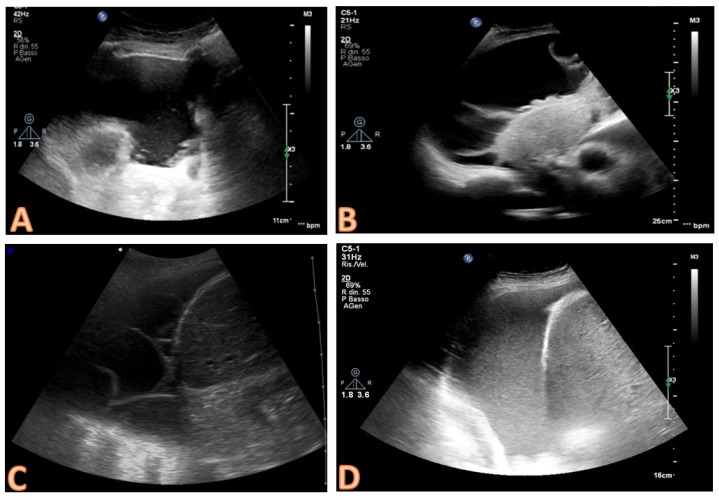
Qualitative evaluation of the effusion. The presence of echogenic material and/or septa within the effusion are suggestive of an exudate. In image (**A**), some echogenic and corpuscular materials are evident in the deep portion of the effusion (non-septated complex); in images (**B**,**C**), the septa are clearly evident (complex septated effusions); in image (**D**), the effusion appears homogeneously echogenic (the case of pleural empyema in the early phase).

The sonographic features of the effusion can be classified on the basis of internal echogenicity and the presence of septs as follows [13,28] (Figure 4):

− anechoic (without echoic images);
− complex non-septated (echogenic material is detected inside the effusion);
− complex septated (floating fibrin strands or septs are found inside the effusions);
− homogenously echogenic.

Complex pleural effusions with septation or internal echogenicity and the effusions with homogeneous echogenicity are always exudates, whereas an anechoic collection is not predictive of the nature of the effusion and could be either an exudate or a transudate [28,29]. 

Knowing approximately the amount of fluid volume and the probable nature of the effusion (transudate or exudates) allows the physician to make an informed decision on benefits and risks of the pleural procedures [13,30].

**Figure 5:**
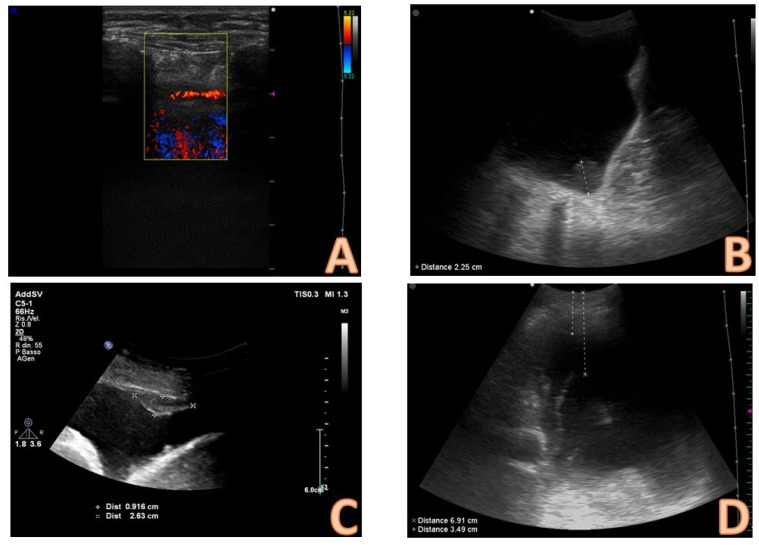
Focused ultrasound evaluation of the needle insertion point. In image (**A**), an anomalous course of an arterial vessel in the intercostal space is evident; therefore, there is a high risk that the needle could injure the vessel, and this finding must induce the operator to vary the choice of insertion point. In image (**C**), a pleural plaque is evident; to insert the needle into the point where this plaque is present can both induce difficulties in inserting the needle and increasing the risk of bleeding from the plaque itself; the needle insertion point should be changed. Image (**B**) demonstrates how a pre-procedural ultrasound evaluation can highlight solid masses at the pleural level; this finding raises the suspicion of a malignant nature of the effusion. In image (**D**), there is an example of the evaluation of the distances between skin and effusion and between skin and lung, which can be done by ultrasound; this evaluation guides the choice of the needle and its insertion depth.

If the pleural effusion is large and complex, it will be necessary to place a drain for therapeutic purposes and/or evaluating video-assisted thoracoscopic surgery (VATS) [30]. Moreover, the operator should evaluate the chest wall and the intercostal space [23] (Figure 5).

The position of the intercostal arteries can vary from one person to another and can be found in a more vulnerable position, going posterior along the single rib, especially in older patients [31]. When arteries move to the middle of the intercostal space are at a greater risk of laceration, and a new site for the procedure should be considered [31]. Furthermore, it is useful to evaluate the presence of any wall masses or pleural thickenings at the needle insertion site, for any risk of bleeding or failure of the procedure [22].

Eventually, it is suitable to measure the insertion depth necessary to reach the pleural fluid as a security control [23]. The angle of the insertion should be reconsidered when the pleural fluid is not obtained at the expected distance [19,22]. The depth necessary to reach the atelectatic lung should be measured and should never be exceeded, and this is particularly relevant for tiny effusions and for overweight or oedematous patients [19,22].

## 5. The Role of CEUS

Contrast-enhanced ultrasound (CEUS) is a well-established and very useful technique for the evaluation of pathological lesions in many body areas [32]. Its application in the pulmonary field is not yet widespread due to some technical difficulties and some pathophysiological principles that are not better clarified (the role of double vascularization at the pulmonary level) [33,34,35]. Despite this, there are several published works showing its usefulness in better defining and characterizing thickenings or nodules/masses at the pleural level [33,36]; in particular, CEUS has proven to be extremely useful in guiding biopsy procedures [36,37]. In the case of thoracentesis, the CEUS method can better highlight some wall formations (the risk of bleeding linked to accidental puncture of the mass), but also clarify whether some formations present within an effusion (for example, some septa or masses) are neoplastic (intake of the contrast medium) or of an “inert” nature such as necrotic material and/or clots [38].

## 6. Thoracentesis Procedure

### 6.1. Position of the Patient

**Figure 6:**
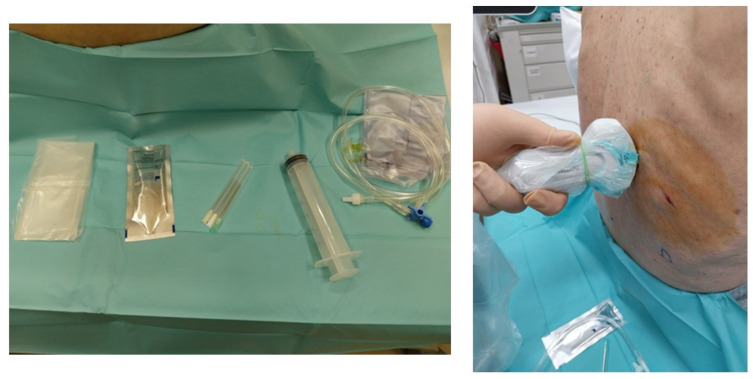
Example of a thoracentesis kit: the kit includes 3 needles of different caliber, a connection system with a 3-way tap, a syringe for aspiration, and a collection bag. On the left side, there is a sterile probe cover and a sterile gel pack. In the best operating setting with a mobilizable and cooperative patient, the patient sits on the edge of the bed and hugs a pillow; this movement allows the intercostal spaces to open more easily. A sterile field with a sterile drape is set up. The operator wears sterile gown and gloves. In this figure, the needle insertion site has already been identified through ultrasound evaluation, and local disinfection is carried out with povidone iodine by using sterile gauze.

To perform a thoracentesis, the patient is usually positioned seated, slightly bent forward and hugging a pillow [22] (Figure 6).

The needle is usually inserted between the posterior axillary line and the mid scapular line, but never under VIII intercostal space to avoid the risk of puncture and therefore accidental injury to the diaphragm and abdominal organs [22]. It is relevant to point out that posteriorly the limits of the costo-phrenic sinus are more caudal than the anterior and lateral portions. If it is not possible to perform the procedure with the patient seated, the other option is that the patient lies in supine position or lateral recumbent position with the side to be drained on top and chest elevated at 30–45° [22,39]. Intercostal scans are obtained over the postero-lateral costophrenic sinus, immediately above the diaphragm, with the probe held longitudinally between the mid and posterior axillary lines [39]. The sinus is reproduced on ultrasound imaging as a transonic triangle bounded by the diaphragm and chest wall. The puncture site is identified at the apex of this triangle [39]. The lines represented by a collapsed lung border and diaphragm create a “V” sign. In this point (called “V point”), it is possible to detect the maximum thickness of pleural effusion. The measure of the maximum depth at the V-point provides for highly efficient ultrasound-guided thoracentesis, and it allows one to estimate the amount of fluid in the pleural cavity [39].

### 6.2. Choice of the Probe

Every probe can be used to perform an ultrasound-assisted procedure [3]. The choice of the probe depends on the setting, the operator experience and preference, the features of the patient, and the information that should be obtained from ultrasound; particularly, the depth of pleural effusion and the presence of parietal lesions or septa are most relevant parameters. 

### 6.3. How to Set the Sterile Field and Local Disinfection

**Figure 7:**
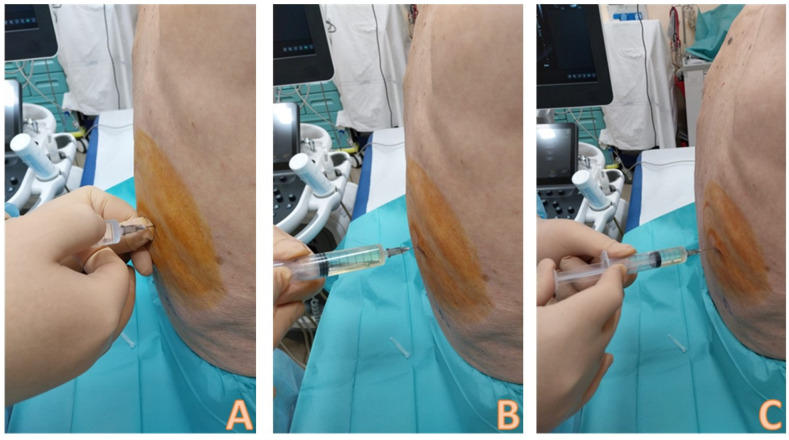
Local anesthesia with lidocaine 1–2%. An ampule of lidocaine is drawn into a 10 mL syringe. In (**A**), the needle insertion phase is visible; the injection is performed at the chosen point and intercostal space. The non-dominant hand palpates the lower rib of the intercostal space; the dominant hand inserts the needle just above the upper edge of the lower rib; the needle is always directed downwards and never upwards to avoid damaging the intercostal artery that runs along the lower edge of the upper rib. The needle must pass the pleura and enter the pleural cavity, so that the local anesthetic can act on the richly innervated pleura; this is demonstrated by the fact that pleural fluid is aspirated into the syringe (**B**). At this point, boluses of 1–2 cc of anesthetic are injected at different points following a backward way to the subcutaneous tissue (**C**).

A sterile field should be set as a first step (Figure 6): This consists in wearing sterile gloves and setting up the sterile field placing a probe cover. Afterwards, the decision about the optimal location point is made and marked on the skin of the patient, and the cutaneous area is cleaned with chlorhexidine or povidone iodine [22]. Local anaesthetic is carried out with 1–2% lidocaine or mepivacaine just above the upper limit of the rib in the intercostal space to avoid puncture of the vascular-nervous bundle [22]. Lidocaine can be injected in the pleural space and in chest wall tissues (Figure 7).

Local anaesthesia is often not required for a simple procedure but should be considered if difficulty attaining the pleural space is likely [12]. In the case of a therapeutic thoracentesis, local anaesthetic should be administered [12].

### 6.4. The Procedure

The thoracentesis follows the procedure performed for local anesthesia. Many thoracentesis kits consist of three cutting needles of different caliber and length; the choice of the type of needle is based on the evaluation of the effusion and the surrounding anatomical structures, as previously mentioned. Currently, Veress needles are available [40]; these needles have been designed to avoid perforation of the organs. They consist of an outer cannula with a sharp tip to flute the mouthpiece and a retractable inner cannula with blunt ends [40]. When the needle punctures the chest wall, the blunt tip is retracted so that the cutting part penetrates into the tissues to cross. Only after reaching the pleural cavity, the spring mechanism pushes the blunt ends beyond the tip of the outer cannula protecting the internal organs from damage.

**Figure 8:**
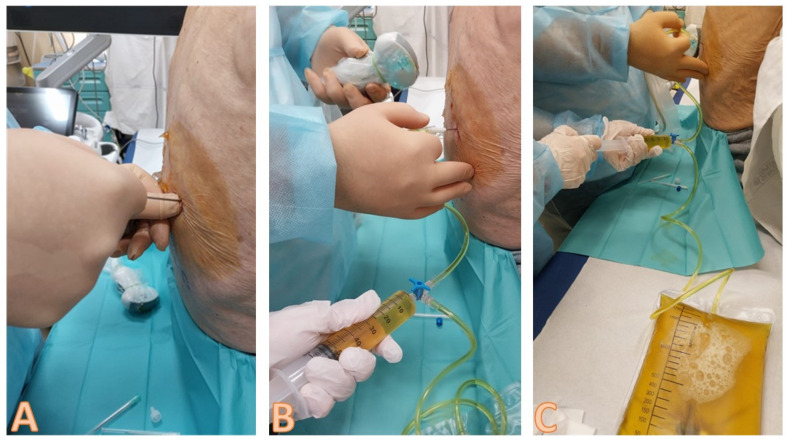
Sequence of images about the insertion of the needle and the execution of the thoracentesis. In image (**A**), the non-dominant hand palpates the lower rib of the chosen intercostal space, and the dominant hand inserts the needle above the upper edge of the rib, always directing it downwards; it should be noted that, as it is an ultrasound-assisted procedure, the probe is not held in the hand when the needle is inserted, but is available to the operator in the sterile field with a sterile probe cover. In (**B**,**C**), the leakage of the pleural fluid through the collection system is highlighted, with a second operator who sequentially aspirates and allows the fluid to flow into the collection bag. Note how the ultrasound probe is taken back into the hand and used to verify the position of the needle and the residual amount of pleural effusion.

The needle is inserted into the established point overcoming the resistance offered by the chest wall and pleura (Figure 8) [19,22]. 

**Figure 9:**
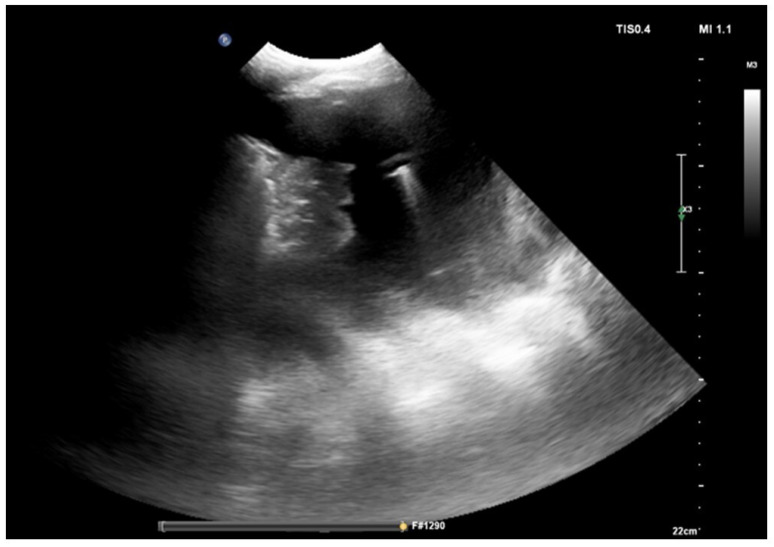
Image highlighting the positioning of the needle inside the effusion during the procedure. The needle is represented as a hyperechoic punctiform image with some posterior artifacts within the anechoic space.

Once in the pleural cavity, the liquid is aspirated via the syringe connected to the collecting system [19,22]. The correct position of the needle must be re-evaluated ultrasonographically, and this can be done multiple times during the method (Figure 9) [19]. 

The probe is positioned on the skin of the patient in the same intercostal space of the needle, just above of it, with an ultrasound scan directed downwards [19]. This is the best way to localize the tip of the needle and control that there is a safe distance during the aspiration of the sample. The needle has a linear hyperechoic structure, surrounded by the pleural effusion, that appears anechoic [19,22]. The liquid is aspirated and then drained into the collection bag. 

## 7. Ultrasound-Guided Thoracentesis

**Figure 10:**
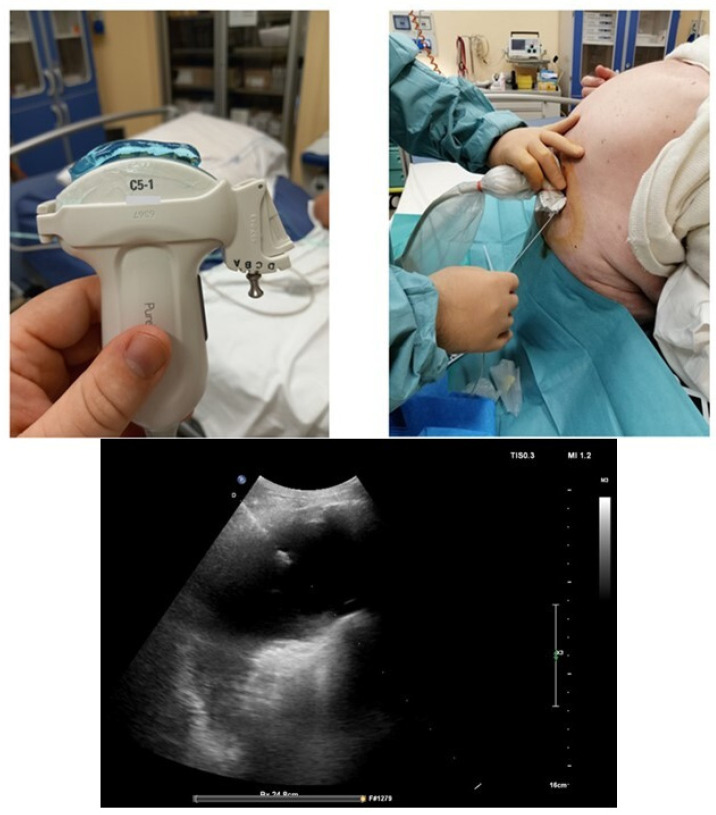
Ultrasound-guided thoracentesis. In this example, we used a guide coupled on a convex probe, which creates a pre-established needle insertion angle. An oblique intercostal scan that allows one to better highlight the pleura and the effusion is performed. The needle is displayed along its entire path, and the exact position of the tip can be observed for the entire duration of the procedure.

The ultrasound-guided procedure has its main indication in the collection of pleural effusions of small volume or complex, and it is usually performed for a diagnostic reason [29,41]. Guidelines do not recommend performing a thoracentesis on a pleural effusion <15 mm; the minimum quote has to be extended for an intercostal space above and one under the point that has been chosen for the needle [12]. There is no difference in the procedure based on the kind of probe that is used during the procedure, but the operator should be aware that the live view identification with a probe could be difficult [10,11,19,22]. Similarly to other invasive procedures, the maneuver can be performed in plane or out-of-plane mode depending on the insertion axis of the needle with respect to the probe; in the specific case of thoracentesis, the probe should be positioned in the intercostal space, thus performing an oblique scan that allows for a good visualization of the pleura and the effusion, and the procedure should be performed in in-plane mode [10,11,19,22]. The out-of-plane mode is equally executable, but the visualization of the needle and in particular the orientation of the needle in the intercostal space is more difficult (with the risk of injury to the intercostal artery) [10,11,19,22]. Furthermore, it is possible to use guides connected to the probe that create a correct coupling between needle and probe and facilitate the maneuver, thereby making thoracentesis more accurate and safer (Figure 10) [10,11,19,22].

## 8. End of the Procedure

There are three indications for suspending the procedure [12]. The first one is the impossibility of draining further fluid. Another eventuality is the presentation of cough or thoracic discomfort by the patient during the thoracentesis [11,12]. The last one is that every single pleural aspiration should be stopped when the volume of 1500 mL is reached [11,12]. Larger drainage volumes were correlated with an increased risk of re-expansion pulmonary edema and pneumothorax. Indeed, there is considerable mortality risk related to re-expansion pulmonary edema and strong evidence supporting minimal complication rates by removing less than 1.5 L [11,12]. A slow removal rate is quite always preferred to favor the gradual lung re-expansion and reduce the risk of re-expansion pulmonary edema [10]. Gravity drainage, hand syringe drainage via a three way stop-cock, and wall suction and vacuum bottle drainage are all options for effusion removal [10]. BTS guidelines suggest connecting the cannula to a three-way tap and fluid withdrawn into the syringe and expelled via the free port of the three-way tap [12]. 

**Figure 11:**
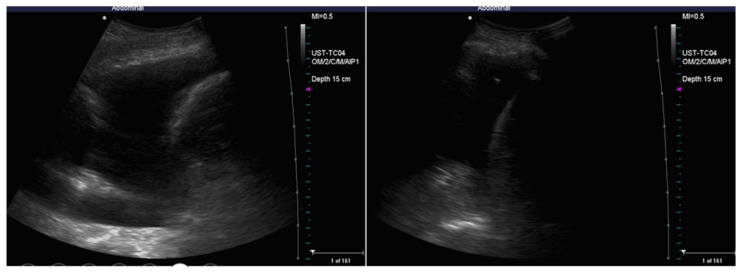
Sequential ultrasound check of the effusion and the position of the needle during thoracentesis. There is a progressive reduction of the anechoic space with progressive re-expansion of the atelectatic lung with the appearance of internal air bronchograms. When the tip of the needle is too close to the re-expanded lung (right figure), the procedure should be stopped.

Furthermore, in our opinion, ultrasound can provide relevant data on when to discontinue the procedure; indeed, when the re-expanding lung comes too close to the needle, it is advisable to suspend the thoracentesis (Figure 11).

**Figure 12:**
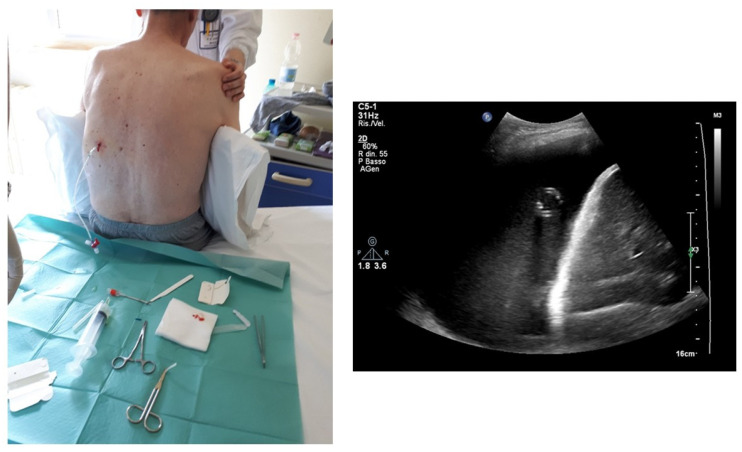
Positioning of 10 Fr caliber pig-tail drainage. The positioning of this device allows gradual removal of the effusion, especially in massive forms, avoiding the development of barotrauma. The correct positioning of the drainage can be verified by ultrasound, viewing a track image with an oval course, corresponding to the tip of the pig-tail.

In case of a diagnostic thoracentesis, BTS guidelines recommend that 20–50 mL of fluid be withdrawn and sent for investigations [12]. Pleural fluid should be submitted for cytological examination in patients with suspected malignant pleural effusion [2]; then, pleural fluid should be sent in both plain and blood culture bottle tubes in patients with suspected pleural infection [2]. When the effusion is very large and/or complex, it is possible to leave a catheter in place using “hybrid” kits that allow a thoracentesis to be quickly converted into a small drainage placement (Figure 12) [22]. 

In patients with suspected complex parapneumonic effusion or empyema, pH analysis is always recommended if available; indeed, fluid pH is an indicator of a high probability of complex parapneumonic effusion/empyema, and it can be used to inform the decision to insert intercostal drainage (Table 1) [2]. Alternatively, it is possible to measure the glycemia level on the pleural fluid, and values <72 mg/dL or <3.3 mmol/L are indicative of empyema [2]. 

These catheters are often of the pig-tail type with a caliber around 8–12 Fr. After chest tube placement, it is not recommended to remove more than 1 L of fluid within the first hour [22]. 

## 9. Complications

The most common complications from pleural aspiration are as follows [5,42]:

− pneumothorax;
− procedure failure;
− pain;
− bleeding or hemothorax (due to a lesion that could occur in the intercostal arteries);
− pulmonary edema;
− “ex vacuo” dyspnea or hypoxemia;
− infection of the site of the procedure;
− abdominal bleeding;
− splenic or diaphragmatic puncture;
− neoplastic seeding (in case of malignant pleural effusion).

It is widely recognized that the physician experience and the use of imaging guidance reduce the rate of post-procedural complications [12,23]. Between the possible complications, the most common one is pneumothorax [43]. The pathophysiology of post-aspiration pneumothorax is likely multifactorial; indeed, some cases are caused by direct lung injury or the introduction of air during the procedure. In other cases, it seems reasonable to affirm that the aspiration of fluid in an unexpandable lung (i.e., for malignant parenchymal lung disease) could produce a significantly low pressure in the pleural space determining an ex vacuo pneumothorax [43,44]. The mechanism by which this damage is generated is not fully known but seems to be related to this “trapped lung” [43]. This uncommon phenomenon is characterized by the introduction of air into the pleural space, a consequence of a vacuum-like negative intrapleural pressure stemming from lung collapse [45]. It is generally observed in patients with bronchial obstructions due to several factors like mucus plugs, aspiration of foreign bodies, and misplacement of the endotracheal tube [46]. This phenomenon is marked by the formation of a thick fibrous peel due to neoplastic cell activity, entrapping the lung and preventing its re-expansion even after the effusion has been drained [47]. 

It is suitable to verify the presence of lung sliding before the execution of the pleural aspiration, and this ultrasound control should be repeated after the thoracentesis [41]. The standardized area to check the presence of pleural sliding is on the hemi-clavicle line of the second intercostal space [48]. It is important to check the presence of sliding before the execution of the thoracentesis, because in certain conditions (i.e., patients with adhesions between parietal and visceral as a consequence of inflammation or neoplastic proliferation in pleural disease) it might not be present, even in the absence of pneumothorax [41]. In that context, it is clear that ultrasound control is useless to exclude the presence of post-procedural pneumothorax. 

Pneumothorax could certainly be excluded if lung sliding or equivalent findings (lung pulse or evidence of B lines) are present [41,48]. Otherwise, the presence of the lung point (the exact area where the normal lung pattern is substituted by the pneumothorax pattern) is 100% specific for the presence of post-operative pneumothorax [41,48].

## 10. Conclusions

− The use of ultrasound increases the success rates of the procedure and significantly reduces the occurrence of complications.
− Any type of probe can ideally be used to perform the procedure.
− Ultrasound provides valuable information on the evaluation of both qualitative and quantitative effusion; in this area, ultrasound is better than chest X-ray and comparable to that of chest CT.
− The choice of the needle insertion point is carried out through ultrasound evaluation, at the point where the effusion is most represented.
− The procedure is preferably performed with an ultrasound-assisted technique.
− The ultrasound-guided procedure is preferable in case of thoracentesis on small and/or complex pleural effusions.
− Ultrasound can optimally visualize the development of a pneumothorax.
− Training on the method must be implemented, in order to disseminate the method and increase its practicability even in complex settings and for non-expert operators.

## Figures and Tables

**Table 1:** Summary of indications to consider intercostal drainage based on pleural fluid pH values. Adapted from Roberts ME., et al. Thorax. 2023 [2]. CPPE: complex parapneumonic pleural effusion; LDH: lactate dehydrogenase.

Pleural Fluid pH ≤ 7.2	Pleural Fluid pH Is >7.2 and <7.4	Pleural Fluid pH ≥ 7.4
High risk of CPPE or pleural infection → intercostal drain should be inserted	Intermediate risk of CPPE or pleural infection → pleural fluid LDH should be measured and, if >900 U/L, intercostal drainage should be considered *	Low risk of CPPE or pleural infection → no indication for immediate drain

* Especially if other clinical parameters support CPPE (specifically ongoing temperature, high pleural fluid volume, and low pleural fluid glucose (<72 mg/dL), septations on computed tomography or ultrasound.

## Data Availability

Not applicable.

## Abbreviations

INR	international normalized ratio
DOAC	direct oral anticoagulant
CrCl	creatinine clearance
BTS	British Thoracic Society
CT	computed tomography
